# How Does Mitochondrial Protein-Coding Gene Expression in *Fejervarya kawamurai* (Anura: Dicroglossidae) Respond to Extreme Temperatures?

**DOI:** 10.3390/ani13193015

**Published:** 2023-09-25

**Authors:** Jing-Yan Wang, Li-Hua Zhang, Yue-Huan Hong, Ling-Na Cai, Kenneth B. Storey, Jia-Yong Zhang, Shu-Sheng Zhang, Dan-Na Yu

**Affiliations:** 1 College of Life Sciences, Zhejiang Normal University, Jinhua 321004, China; 2 Taishun County Forestry Bureau, Wenzhou 325000, China; 3 Department of Biology, Carleton University, Ottawa, ON K1S 5B6, Canada; 4 Key Lab of Wildlife Biotechnology, Conservation and Utilization of Zhejiang Province, Zhejiang Normal University, Jinhua 321004, China; 5 Zhejiang Wuyanling National Nature Reserve, Wenzhou 325500, China

**Keywords:** *Fejervarya kawamurai*, mitochondrial genome, phylogeny, mitochondrial gene transcript level, low- and high-temperature stress

## Abstract

**Simple Summary:**

The tropical and subtropical paddy frog, *Fejervarya kawamurai* (Anura: Dicroglossidae), is a common and widespread species in Asia. Amphibians can adapt to small changes in climate, but extreme temperature changes can lead to metabolic abnormalities affecting numerous cell functions. Damage or disruption of mitochondrial respiratory chain complexes can be lethal. The present study characterizes the mitochondrial genome of *F. kawamurai* to evaluate its evolutionary relationship within Dicroglossidae and to analyze the effects of extreme temperature change on mitochondrial gene expression of proteins involved in oxidative phosphorylation.

**Abstract:**

Unusual climates can lead to extreme temperatures. *Fejervarya kawamurai*, one of the most prevalent anurans in the paddy fields of tropical and subtropical regions in Asia, is sensitive to climate change. The present study focuses primarily on a single question: how do the 13 mitochondrial protein-coding genes (PCGs) respond to extreme temperature change compared with 25 °C controls? Thirty-eight genes including an extra tRNA-Met gene were identified and sequenced from the mitochondrial genome of *F. kawamurai*. Evolutionary relationships were assessed within the Dicroglossidae and showed that Dicroglossinae is monophyletic and *F. kawamurai* is a sister group to the clade of (*F. multistriata* + *F. limnocharis*). Transcript levels of mitochondrial genes in liver were also evaluated to assess responses to 24 h exposure to low (2 °C and 4 °C) or high (40 °C) temperatures. Under 2 °C, seven genes showed significant changes in liver transcript levels, among which transcript levels of *ATP8*, *ND1*, *ND2*, *ND3*, *ND4*, and *Cytb* increased, respectively, and *ND5* decreased. However, exposure to 4 °C for 24 h was very different in that the expressions of ten mitochondrial protein-coding genes, except *ND1*, *ND3,* and *Cytb*, were significantly downregulated. Among them, the transcript level of *ND5* was most significantly downregulated, decreasing by 0.28-fold. Exposure to a hot environment at 40 °C for 24 h resulted in a marked difference in transcript responses with strong upregulation of eight genes, ranging from a 1.52-fold increase in *ND4L* to a 2.18-fold rise in *Cytb* transcript levels, although *COI* and *ND5* were reduced to 0.56 and 0.67, respectively, compared with the controls. Overall, these results suggest that at 4 °C, *F. kawamurai* appears to have entered a hypometabolic state of hibernation, whereas its mitochondrial oxidative phosphorylation was affected at both 2 °C and 40 °C. The majority of mitochondrial PCGs exhibited substantial changes at all three temperatures, indicating that frogs such as *F. kawamurai* that inhabit tropical or subtropical regions are susceptible to ambient temperature changes and can quickly employ compensating adjustments to proteins involved in the mitochondrial electron transport chain.

## 1. Introduction

As one of numerous pervasive environmental factors, temperature change greatly affects the physiology of vertebrate species, including amphibians [1]. Amphibians are vulnerable to a wide range of temperature variations since their lifecycle often involves both aquatic and terrestrial settings [2]. Adverse ambient temperatures can cause animals to retreat into states such as dormancy, aestivation, or hibernation [3]. Many species that can survive under extreme ambient temperatures for long periods need to greatly slow down their metabolism and enter a state of hypometabolism [4,5,6,7,8]. Hibernating animals in low-oxygen environments benefit from strong metabolic rate suppression that effectively extends the time that their endogenous fuel reserves (e.g., carbohydrates and lipids) can support viability [9]. At the genetic level, hypometabolism can manifest as the gene transcription level changes. Also, the overproduction of reactive oxygen species (ROS) under the influence of extreme temperatures can harm important biological molecules including proteins, DNA, and lipids, impairing cellular functions and leading to oxidative stress [7,10,11,12]. To better adapt to a temperature that is not conducive to active life and survive as long as possible under adverse environments, vertebrates can adjust their metabolism and adopt specific behaviors, such as dormancy, enzyme activities, and internal environment regulation. One example of a significant triggering factor for hibernation is temperature [13,14]. Hibernation is an important aspect of amphibian life history because it serves as a protective mechanism against frigid temperatures [15]. For some frost-resistant amphibians, lowering their metabolic rate can help them survive longer in a dormant state. With freeze–thaw episodes, however, freeze-tolerant amphibians may experience somewhat higher energy needs in the subnivium [16]. Moreover, in winter, freeze-tolerant organisms typically amass cryoprotective osmolytes [17]. For example, freezing temperatures trigger wood frogs (*Rana sylvatica*) to synthesize ice-nucleating proteins (INPs) and greatly increase glucose and urea concentrations to act as cryoprotectants [18,19,20]. Other species, such as gray treefrogs (*Dryophytes versicolor*), accumulate glycerol instead [21,22], which is the cryoprotectant of choice for most invertebrate species. Similar metabolic behaviors can be observed in other amphibians that inhabit comparable natural habitats, but the carbohydrate protectant varies from species to species.

Amphibians are equipped with a special set of biochemical and physiological mechanisms to adapt to variations in ambient temperature [23]. For example, they can generate chemicals that enhance stress resistance and modify mitochondrial transcript levels of protein-coding genes (PCGs) to adapt to varying ambient temperatures [24,25]. During oxidative phosphorylation (OXPHOS), mitochondria perform a major role in producing ATP [26,27]. Amphibian mitochondria are also essential for adaption to ambient temperature change [28]. In harsh environments, animals reduce their metabolic rate by diminishing their ATP demand and endogenous fuel consumption [29,30,31,32,33,34,35,36]. The metabolic rate of ectotherms is temperature-dependent, with a sharp drop occurring at colder temperatures [37]. Numerous studies have shown that temperature variations can affect how PCGs are expressed in mitochondrial respiratory chain complexes [38,39,40,41,42,43,44,45] in order to facilitate the reorganization of metabolism under shifting ambient temperatures [40]. However, the level of gene expression varies from species to species. For instance, after 24 h in a cryogenic (frozen) state, transcript levels of mitochondrial genes 16S RNA, *ATP6/8*, and *ND4* of *Rana sylvatica* were dramatically upregulated [46]. Conversely, relative transcription of the *COI* gene in *Dryophytes versicolor* mitochondria was reduced following freezing stress [47]. The preponderance of mitochondrial PCGs in diverse organs of the tiger frog (*Hoplobatrachus rugulosus*) were all downregulated under low-temperature stress [39]. This sensitivity of gene expression to temperature or climate can affect the amphibian life cycle and the metabolism of ROS [48,49]. Regardless of the changes in expression levels, such changes indicate that mitochondria action is closely integrated into biological cold and/or freeze tolerance.

Over the past century, the Earth’s average atmospheric temperature has warmed by 0.6 °C [50,51] and is expected to climb 1.8–4.0 °C before the century is out [52,53]. Even though there is no conclusive evidence that the rate of global warming is necessarily increasing, it is undeniable that extreme climate events caused by global warming will lead to the extinction of multiple species [54,55,56]. Due to global warming, amphibians could become the most endangered animals on the planet [57], especially those species in tropical and subtropical areas [58]. Extreme high temperatures can lead to dysregulation of metabolic pathways, damage to cell structures, and even death when temperatures exceed the critical maximum for each species [9,59]. To prolong survival in extreme heat, cells will take special metabolic actions to eliminate harmful substances induced by high temperatures. An overexpression of ROS might stimulate the upregulation of HSP gene expression [60,61,62]. Production of heat shock proteins (HSPs) can protect protein structure and prevent aggregation of denatured proteins in their capacity as molecular chaperones [63,64]. Not just heat, heavy metal ions, oxidative stress, and arsenite are also some of the stressors that trigger the production of heat shock proteins [65,66,67]. Heat can induce oxidative stress and trigger the production of antioxidant enzymes [68,69]. Additionally, high temperatures are associated with enhanced oxygen consumption. For instance, elevated temperatures enhanced resting oxygen consumption in *Thamnophis elegans* [70], but *Python regius* did not plateau at temperatures approaching critical thermal maximum (CT_MAX_) [71]. Although few studies have directly elucidated the effects of high-temperature stress on mitochondrial genome expression levels, as an essential component of cellular metabolism, mitochondria should play a crucial role in this process. Indeed, the heart of rainbow trout (*Oncorhynchus mykiss*) held at high temperatures showed a drop in phosphorylation and uncoupled respiration ratios, and phosphorylation gradually decreased as the temperature increased, whereas mitochondria proton permeability increased, meaning more proton leakiness [72]. The study also found that when approaching the thermal limit temperature, complex I (NADH dehydrogenase) activity decreased [72].

The current research on amphibian responses to temperature change is limited. Few studies have focused on amphibians that live in subtropical and tropical regions and hibernate. The rice frog, *Fejervarya kawamurai*, a common species of paddy frog, belongs to the anuran family Dicroglossidae [73,74]. The complete mitochondrial genome sequence and phylogenetic relationship of *F. kawamurai* from Guizhou province, China, have been published by Cheng et al. [75]. It is a member of the *Fejervarya limnocharis* complex with *F. kawamurai* living mainly on the Japanese mainland, the central Ryukyu islands of Japan, and southern China [74]. Thus, *F. kawamurai* is a good representative for studying subtropical and tropical amphibian responses to extreme cold or heat stress.

The frequency of exceptionally hot or cold days may rise as a result of human intervention, which is anticipated to have a particularly significant effect on ectothermic species [76,77]. The present study investigated the phylogenetic relationship of *F. kawamurai* within Dicroglossidae and the relative expression of mitochondrial PCGs in response to cold or heat exposure using the mitochondrial genome. The major goal of the study was to determine how the 13 PCGs differ in expression patterns with temperature change by comparing frogs held at 2 °C, 4 °C, 25 °C, or 40 °C.

## 2. Materials and Methods

### 2.1. Animal Treatments

Twenty-four similar-sized adult frogs (*Fejervarya kawamurai*) were collected on 5 September 2021 from a paddy field in Guangzhou, Guangdong Province, China (113°20′39′′ E, 23°3′52′′ N). Upon arrival in the lab, all animals were bathed in a tetracycline solution and then held and fed in a plastic incubator for a week at 25 °C. The frogs were then randomly distributed into four groups of six samples. The frogs in the control group were kept in a plastic box with a moist towel for 24 h at 25 °C. Two groups were exposed to low-temperature stress for 24 h at 2 °C or 4 °C. The fourth group was exposed to high-temperature stress at 40 °C for 24 h. Subsequently, all frogs were euthanized by pithing, followed by quick liver dissection and freezing in liquid nitrogen. Organ samples were then held at −80 °C until use.

### 2.2. DNA Extraction, PCR, and Sequencing

Using a Column Animal Genomic DNA Purification Kit (Sangon Biotech Company, Shanghai, China), total genomic DNA from *F. kawamurai* was retrieved from a clipped toe. Fourteen pairs of common primers were modified using the techniques of Yu et al. [78], Liu et al. [79], and Huang et al. [80] referring to the published sequence [75] in the NCBI and common frog primers devised by Zhang et al. [81,82]. PCR and LA-PCR were amplified using the methods described by Zhang et al. [83]. Sangon Biotech Company (Shanghai, China) sequenced all PCR products utilizing the bi-directional primer-walking method.

### 2.3. Sequence Assembly and Analysis

DNASTAR Package v.6.0 [84] was used to manually evaluate and assemble all sequences. MITOS2 (http://mitos2.bioinf.uni-leipzig.de/index.py, accessed on 11 May 2022) [85] detected all genes. Mega 7.0 [86] was used to compare the position of all genes to those of closely related species using sequences downloaded from GenBank. All tRNA genes were further evaluated by their cloverleaf secondary structure utilizing tRNA-scan SE 2.0 (http://lowelab.ucsc.edu/tRNAscan-SE/, accessed on 11 May 2022) [87] or by comparing their sequences to those of other species. The CG View online server v.1.0 (https://cgview.ca/, accessed on 15 May 2022) [88] generated complete mitochondrial genome maps of *F. kawamurai*. PhyloSuite v.1.2.2 [89] calculated the CG and AT skews. Adobe Illustrator 2020 was used to illustrate the relative synonymous codon usage (RSCU) of PCGs.

### 2.4. Molecular Phylogenetic Analyses

To investigate the phylogenetic relationship among Dicroglossidae, a dataset including the complete mitochondrial genomes from other anuran groups (*Limnonectes*, *Fejervarya*, *Hoplobatrachus*, *Phrynoderma*, *Nanorana*, and *Quasipaa*) [38,75,79,90,91,92,93,94,95,96,97,98,99,100,101,102,103] as well as an outgroup species belonging to *Occidozyga* [104] was created (Table 1). Since *Hoplobatrachus rugulosus* contains two distinct *ND5* genes [78,93], *ND5* was not used to construct the phylogenetic trees. Finally, phylogenetic analyses were conducted using concatenated sequences of the 12 PCGs of complete mitochondrial genomes. DAMBE v.4.2 [105] was used to test substitution saturation using the 12 PCGs nucleotide sequences dataset. Since the first, second, and third codon positions were not saturated, the first, second, and third codons of the 12 PCGs (the PCG123 dataset) were used for phylogenetic analyses. MAFFT v.7 [106] was used to align the 13 PCG nucleotide sequences and Gblock 0.91b [107] was used to detect the conservative region using the default configuration. PhyloSuite v.1.2.2 [89] was used to concatenate the resulting alignments and Geneious v.8.1.6 [108] was used to convert it. PartitionFinder v.2.2.1 [109] was employed to select the best substitution model of the PCG123 dataset for Bayesian inference (BI) and maximum likelihood (ML) analyses. The PCG123 dataset yielded a total of nine partitions, the results of which are displayed in Table S1. The phylogenetic analysis employed the GTR + I + G model. In MrBayes v.3.2 [110], BI analysis was conducted for 10,000,000 generations until the average standard deviation of Bayesian split frequencies fell below 0.01. RaxML v.8.2 software [111] was used to perform ML analysis with rapid inference evaluation for each node under 1000 ultrafast replications. The first 25% of the generations were burned-in to improve phylogenetic analysis. When the value of the average standard deviation of the split frequency was stable and the balance was less than 0.01, the tree was extracted.

### 2.5. RNA Extraction and cDNA Synthesis

Total RNA of *F. kawamurai* was extracted from frozen liver samples of the control (25 °C), 24 h low-temperature (2 °C and 4 °C), and 24 h high-temperature (40 °C) conditions using a TaKaRa MiniBEST Universal RNA Extraction Kit (Takara, Japan). After 15 min of electrophoresis on a 1% agarose gel at 135 V and 120 mA, the samples were stained with Goldview. Sharp bands for 28 S and 18 S ribosomal RNA confirmed RNA integrity [112]. RNA was stored at −80 °C until usage. Take3 apparatus (BioTek Instruments Inc., Winooski, VT, USA) was used to assess RNA content and quality at 260 nm and 280 nm. Following the instructions of a PrimeScript™ RT Master Mix kit (Takara, Japan), 500 ng of RNA-containing sample volumes were gently mixed for reverse transcription. Reactions were carried out under the following conditions: reverse transcription at 37 °C for 15 min and then inactivation of the reverse transcriptase at 85 °C for 5 s.

### 2.6. RT-qPCR Primer Design

According to the mitochondrial gene sequence of *F. kawamurai*, MegAlign (DNASTAR) and Primer Premier 6.0 software (Premier Biosoft International, Palo Alto, CA, USA) were used to build reverse transcription–quantitative polymerase chain reaction (RT-qPCR) primers. *β-actin* served as the reference gene [113,114]. PCR primers for *β-actin* were taken from Jin et al. [39]. Table 2 lists the RT-qPCR primers synthesized by Shanghai Biotechnology Company (Shanghai, China). The length of amplicons varied from 120 to 150 bp, melting temperatures were designed between 50 °C and 55 °C, and primer lengths were between 18 and 22 bp.

### 2.7. Relative mRNA Quantification

A StepOnePlus™ Real-Time PCR System (Life Technologies, Carlsbad, CA, USA) was used to quantify transcript levels of the 13 PCGs. Standard curves and gene quantification primers were tested using serial dilutions of the control group of pooled cDNA. Each sample was mixed with 10 µL SYBR Premix Ex Taq II (2×), 0.4 µL ROX Reference Dye (50×), 0.8 µL forward and reverse primers (10 µM), 6 µL ddH_2_O, and 2 µL RT reactants (cDNA) for RT-qPCR. Primers and genes were used for three technical replicates with conditions of 95 °C for 30 s for denaturation followed by 40 cycles of 95 °C for 5 s and 55 °C for 30 s. Relative mRNA quantification was calculated by dividing the first target gene quantity by the starting gene amount for each sample.

### 2.8. Data Analysis

Each experimental condition involved four unique biological replicates for each gene, and data were reported as mean expression ± SE. The relative levels of mRNA transcripts were determined using the 2^−ΔΔCt^ method and standardized to the *β-actin* gene. All data were analyzed using Statistical Program for Social Sciences 22.0 software (SPSS, Inc., Chicago, IL, USA). Grubbs (Extreme Studentized Deviate Test) eliminates outliers and has 95% credibility. Student’s *t*-test was used to compare gene transcript levels in livers from the control and experimental groups, with *p* < 0.05 considered as a significant difference [115]. Data were graphically presented Using Origin 2021 software (Origin Lab).

## 3. Results

### 3.1. General Features of the F. kawamurai Mitogenome

**Figure 1:**
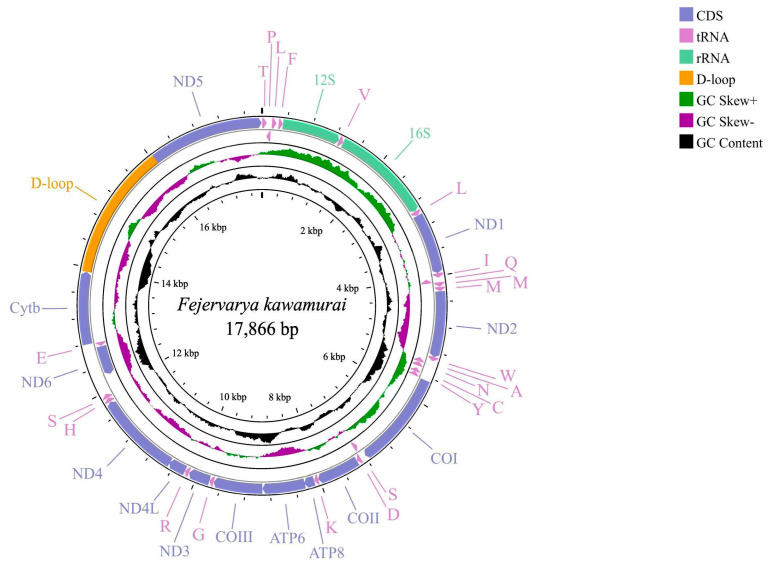
Circular visualization maps of the complete mitochondrial genome of *F. kawamurai*. The circles from the outside to the inside show the gene map (PCGs, rRNAs, tRNAs, and the AT-rich region), the GC content, and the GC skew, respectively. Among them, the genes outside the map are coded on the majority strand (J-strand), whereas the genes inside the map are coded on the minority strand (N-strand).

**Figure 2:**
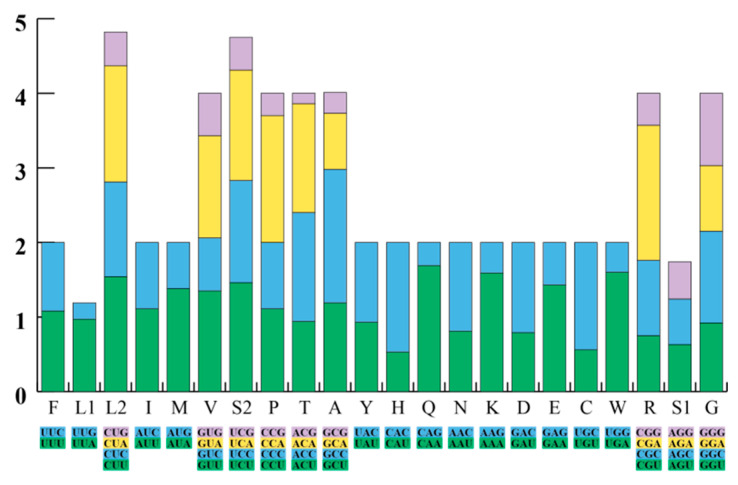
The relative synonymous codon usage (RSCU) of the mitochondrial genomes of *F. kawamurai*. Acronyms stand for different amino acids. The *x*-axis represents all codons used and different combinations of synonymous codons. The RSCU values are listed on the *y*-axis.

The *F. kawamurai* mitochondrial genome was 17,866 bp and is available in GenBank with accession number OQ633008. The circular mitogenome encoded 13 PCGs, 23 tRNAs (including an extra tRNA-Met), 2 rRNA genes (12S rRNA and 16S rRNA), and a D-loop between *Cytb* and *ND5* (Figure 1 and Figure S1). The L strand encodes *ND6* and 8 tRNAs, whereas the H strand encodes the remaining genes. Table 3 shows the features of all genes in the mitochondrial DNA. The AT skew, GC skew, and A + T content of the whole genome, PCGs, rRNAs, and tRNAs were calculated (Table 4). Moreover, mitochondrial DNA is extraordinarily compact and parsimonious [116]. Two pairs of H-strand genes, *ATP8*-*ATP6* and *ND4L*-*ND4,* had open reading frame (ORF) overlaps. Some PCGs share nucleotides with nearby tRNA genes. Using Tandem Repeats Finder v.4.09 [117], the tandem repeats between tRNA-Ser and *ND6* included the tRNA-Ser downstream sequence and the *ND6* upstream sequence, and a gap between them was found. Figure 2 displays the Relative Synonymous Codon Usage (RSCU) of the 13 mitochondrial PCGs from *F. kawamurai*. Excluding stop codons, the mitochondrial genome of *F. kawamurai* encoded 3742 amino acids.

### 3.2. Phylogenetic Relationships of F. kawamurai

**Figure 3:**
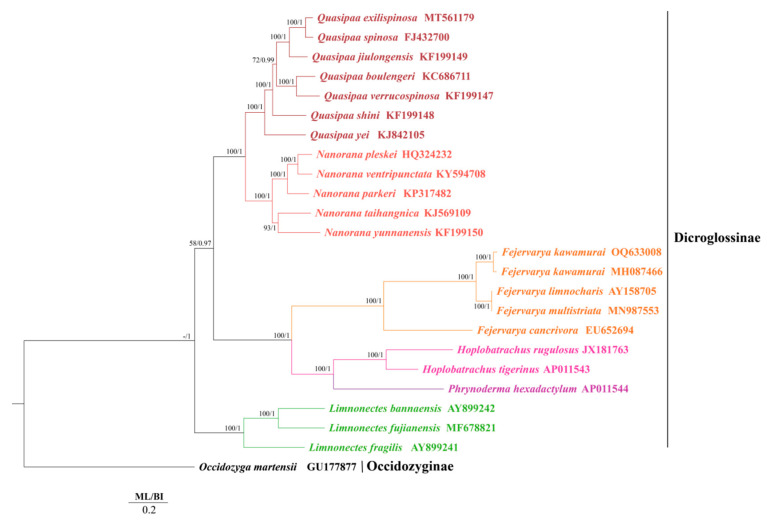
Phylogenetic relationships among 23 species (24 sequences) of Dicroglossidae based on the nucleotide dataset of the 12 mitochondrial protein-coding genes. *Occidozyga martensii* was used as the outgroup. The numbers above the branches specify bootstrap percentages from ML (**left**) and posterior probabilities as determined from BI (**right**). The GenBank accession numbers of all species are shown in the figure. Different colors represent different genera.

BI and ML trees based on entire mitogenome sequences had identical topologies and bootstrap and posterior probabilities were well supported internal nodes (Figure 3). In both BI and ML trees, *Occidozyga martensii* was used as the outgroup. In the subfamily Dicroglossinae, *F. kawamurai* was a sister clade to the clade of (*F. multistriata* + *F. limnocharis*), then *F. cancrivora* was a sister clade of ((*F. multistriata* + *F. limnocharis*) + *F. kawamurai*). *P. hexadactylum* was a sister clade of (*H. rugulosus* + *H. tigerinus*) and then clustered in a clade with (*F. cancrivora* + ((*F. multistriata* + *F. limnocharis*) + *F. kawamurai*)), later clustered in a clade with (*Quasipaa* + *Nanorana*), and finally clustered in a clade with (*Limnonectes fragilis* + (*Limnonectes bannaensis* + *Limnonectes fujianensis*)).

### 3.3. Transcript Levels of Protein-Coding Mitochondrial Genes

**Figure 4:**
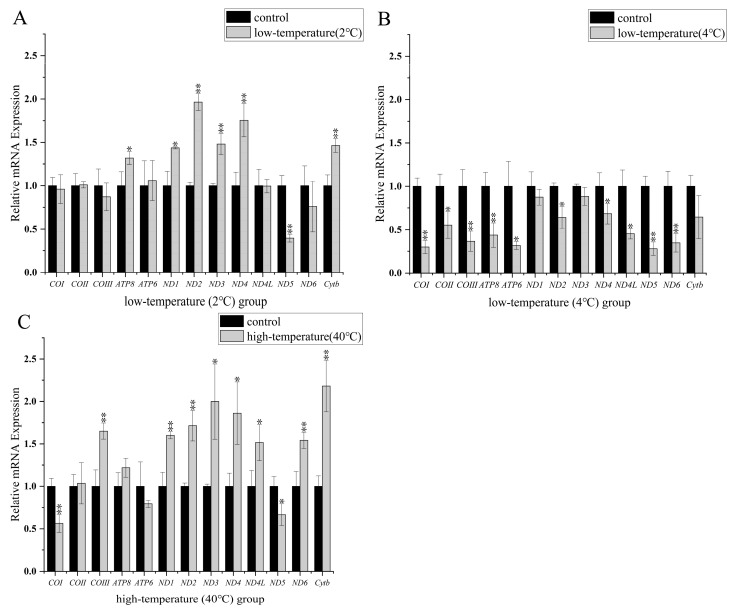
The steady-state transcript levels of 13 protein-coding genes of *F. kawamurai* in response to different temperature stresses. The *x*-axis shows gene name and the *y*-axis shows gene steady-state transcript levels. Black columns show controls (25 °C) standardized to 1.0; the hatched columns show the corresponding experimental group ((**A**): 2 °C; (**B**): 4 °C; (**C**): 40 °C). Asterisks indicate significantly different expression: (*, *p* < 0.05) and (**, *p* < 0.01).

*RT*-qPCR was used to compare hepatic transcript levels of the 13 PCGs in *F. kawamurai* under control (25 °C), low-temperature (2 °C and 4 °C), and high-temperature settings. Gene v mean values were standardized to 1.0 ± SEM in the control group and values for other groups were expressed relative to the control group (Figure 4).

Compared with the control group (25 °C), liver mitochondrial gene transcript levels of *ATP8*, *ND1*, *ND2*, *ND3*, *ND4*, and *Cytb* increased by 1.32 ± 0.07, 1.44 ± 0.01, 1.96 ± 0.10, 1.48 ± 0.12, 1.76 ± 0.19, and 1.46 ± 0.08 fold, respectively, when assessed at 2 °C. Only *ND5* gene expression decreased to a value of 0.40 ± 0.05 compared with the control. The expression levels of the remaining 6 PCGs were not significantly different between the control and low-temperature states (Figure 4A). After 4 °C low-temperature stress, mitochondrial gene transcripts of *COI*, *COII*, *COIII*, *ATP6*, *ATP8*, *ND2*, *ND4*, *ND4L*, *ND5*, and *ND6* were markedly lower, decreasing to values 0.30 ± 0.08, 0.56 ± 0.15, 0.37 ± 0.12, 0.32 ± 0.05, 0.44 ± 0.14, 0.64 ± 0.13, 0.68 ± 0.12, 0.45 ± 0.06, 0.28 ± 0.08, and 0.35 ± 0.11 fold compared with the control group. However, *ND1*, *ND3*, and *Cytb* transcripts showed no significant differences between the control and 4 °C groups (Figure 4B). Compared with the low-temperature stress groups, the 40 °C group showed transcript levels of *COIII*, *ND1*, *ND2*, *ND3*, *ND4*, *ND4L*, *ND6*, and *Cytb* that were significantly elevated by 1.65 ± 0.10, 1.06 ± 0.04, 1.72 ± 0.18, 2.00 ± 0.44, 1.86 ± 0.37, 1.51 ± 0.21, 1.54 ± 0.10, and 2.18 ± 0.30 fold, respectively. However, *COI* and *ND5* transcript levels were lowered to 0.56 ± 0.11 and 0.67 ± 0.13, respectively, compared with the control group (Figure 4C).

## 4. Discussion

### 4.1. Phylogenetic Relationships

Most of the phylogenetic results were similar to previous studies [75,82,118]. According to the results, the evolutionary position of *F. kawamurai* in Dicroglossidae was again clarified. In phylogenetic trees, Occidozygini (Occidozyginae) was observed to be a basal clade to Dicroglossinae, which is basically consistent with previous reports [99]. In the subfamily Dicroglossinae, *Fejervarya* is a sister clade to (*Hoplobatrachus* + *Phrynoderma*). However, this study found that *Limnonectes* was a sister clade of ((*Quasipaa* + *Nanorana*) + (*Fejervarya* + (*Hoplobatrachus* + *Phrynoderma*))), which was inconsistent with the results of Cheng et al. [75], Zhang et al. [82], and Yu et al. [99]. This discrepancy was due to the fact that different datasets were used to reconstruct the evolutionary relationship. Furthermore, due to the diversity of species having morphological variation, the classification of *Fejervarya* was controversial in early studies [119]. In particular, *Fejervarya limnocharis* is now considered to be a complex species, with four species currently in it: *Fejervarya kawamurai*, *Fejervarya sakishimensis*, *Fejervarya multistriata*, and a monophyletic group from southeastern and eastern Taiwan (*Fejervarya* sp.) [74,120]. Therefore, the phylogenetic relationship of *Fejervarya* still needs to be further investigated.

### 4.2. Mitochondrial Transcript Level Analyses at Low Temperature

To investigate the metabolic activities of organisms, it is impossible to completely isolate them from their native habitat’s natural environment. For instance, *Drosophila melanogaster* inhabiting tropical regions exhibits greater tolerance to elevated temperatures compared with those residing in higher latitudes. These variations in temperature tolerance are believed to be associated with dissimilarities in the mitochondrial genome [41]. Similarly, amphibians living in subtropical areas are much less tolerant to low temperatures than those living in higher latitudes, and this seems to be reflected at the mitochondrial genome level. How does the *F. kawamurai* species, if captured in Guangzhou where winter temperatures rarely drop below freezing, cope with the uninhabitable extreme cold? This low-temperature experiment was designed at a lower temperature than 4 °C, and the result found that there was a difference between them. At 4 °C, transcript levels of 10 PCGs from *F. kawamurai* were reduced significantly compared with the 25 °C controls. Nevertheless, at 2 °C, the expression of 6 PCGs increased. Compared with the 25 °C control group, *ND5* transcription was reduced considerably at 2 °C and 4 °C. This might suggest a rate-limiting role for the *ND5* protein in mediating mitochondrial activity at cold temperatures.

Indeed, physiological adaptation for cold hardiness could depend substantially on temperature-induced changes in gene expression [121], particularly at the mitochondrial level [37]. Proteins encoded by mitochondrial DNA participate in the respiratory chain complexes I, III, IV, and V. Complex I, type I NADH dehydrogenase, is essential to cellular metabolism. The tricarboxylic acid cycle and other physiological functions require reducing equivalents from NADH oxidation to NAD^+^ [122]. Cytochrome b with two membrane-side quinone-binding sites, is a subunit of complex III encoded by mitochondria [123]. ATPase (complex V) is closely related to ATP production during oxidative phosphorylation. All of these complexes are associated with proton transfer and oxidative phosphorylation. Ectotherms can often survive extreme settings, particularly cold temperatures, by allowing their metabolic rate to decrease in proportion to declining environmental temperatures [4,5,6,7,8,33,34,35,36,124].

It has been demonstrated that poikilotherms can increase their duration of survival under stressful conditions by slowing their metabolic rate and thereby lowering the drain on endogenous fuel supplies caused by a need to generate ATP [33,34,35,36,124]. This low metabolic state can effectively help amphibians survive the long winter. For this purpose, amphibians must reduce oxygen usage by altering mitochondrial metabolism and affinity, membrane permeability, and cellular electrochemical gradients [125]. The declining expression level of electron transport chain subunits is evidence of hypometabolism. For example, exposure to chilling decreased *COI* transcript levels in gray treefrogs, *D. versicolor* [47]. The majority of mitochondrial genes in various organs of *H. rugulosus* were downregulated under low-temperature conditions [39]. In addition, low ambient temperature affects amphibian activity and foraging [126]. When subjected to cold exposure, a lower resting metabolic rate in *Bufo marinus* by hypometabolism was found [37]. Therefore, it is reasonable to speculate that the significant downregulation of some mitochondrial gene transcription levels in *F. kawamurai* at 4 °C indicates that it has entered a low metabolic dormant state.

However, at 2 °C, the transcript levels of 6 PCGs were significantly increased, affecting mitochondrial respiratory chain complexes I, III, and V. This phenomenon might be related to hepatocyte glycogen breakdown and gene expression for cryoprotectant or antifreeze production [16,24]. The conversion of glycogen into the carbohydrate protectant required by the organism requires a certain amount of ATP to supply energy, and the original production capacity in the low metabolic state is not enough to ensure this additional energy demand. Moreover, ROS, including hydroxyl radicals, superoxide, and peroxyl radicals, could increase in concentration in a frigid environment [11] and, in excess, oxidative stress can harm vital biological molecules [127,128,129,130], including subunits of mitochondria. The respiratory chain is oxidized by ROS [131], leading to a decrease in electron flow and ATP synthesis [132]. These markedly upregulated genes are associated with proton transport and ATP production. Therefore, maintaining essential metabolism in the face of oxidative damage caused by ROS might be responsible for the increased level of mitochondrial PCG expression at very low temperatures.

### 4.3. Mitochondrial Transcript Level Analyses at High Temperature

Mitochondrial dysfunction at high temperatures may be the cause of an animal’s upper thermal limitations. Most liver mitochondrial PCGs were upregulated by high-temperature stress. Indeed, at 40 °C, transcript levels of 7 PCGs increased significantly in *F. kawamurai* liver. However, *COI* and *ND5* gene transcript levels were significantly reduced.

The thermal limit of an organism is correlated with the normal function of the mitochondrial respiratory chain. Mitochondrial proton leakage at higher temperatures appears to be larger than usual, implying that inefficient proton circulation rises, lowering the effective P/O ratio [72,133]. At elevated temperatures, this will inevitably pose a challenge to the energy supply of cells. Heat-induced increases in ROS can damage organelle membranes [68,69] and heat can also inactivate complexes I, III, and IV of the electron transport chain and destroy them by causing mitochondrial oxidative injury [134]. Such injury can interfere with the normal coupling of the respiratory chain response, rendering mitochondria incapable of producing sufficient energy. In addition, according to the oxygen- and capacity-limited thermal tolerance (OCLTT) theory, the oxygen supply capacity of the organism at thermal limits is insufficient to meet the needs of aerobic respiration [135,136,137,138]. Collectively, the significantly increased expression levels of a number of mitochondrial genes may serve to compensate for the insufficient energy provided by the mitochondrial respiratory chain in response to high-temperature duress. OCLTT also integrates protective mechanisms, including chaperones, anaerobic metabolism, and antioxidative defenses. Therefore, SOD, CAT, GPx, and some non-enzymatic antioxidants produced in heat conditions may be connected to the expression of mitochondria genes [62,139,140]. In addition, the change trend of transcription levels of *COI* and *COIII*, which belong to complex IV [141], is different. Consequently, high ambient temperature can affect steady-state transcript levels of electron transport chain PCGs differently. Taken together, the variation in mitochondrial gene transcript level induced by high-temperature stress could be associated with compensating the activity of mitochondrial respiratory chain complexes and modulating inner mitochondrial membrane proton permeability.

### 4.4. Characteristics of ND5 Gene Expression

Compared with the control group, no matter whether under high- or low-temperature conditions, *ND5* transcripts consistently showed a significant reduction in liver. The *ND5* gene encodes the ND5 protein that is a long horizontal α-helix in the hydrophobic arm of complex I. Its length varies from species to species [142]. The α-helix containing transmembrane helices (TMH) at the C-terminus of ND5 can provide structural stability by clamping the two proton pumping modules [143,144]. In a study of low-temperature stress in *H. rugulosus* from China and Thailand, two identical *ND5* genes in *H. rugulosus* from China exhibited significant differences in expression, whereas two different *ND5* genes in *H. rugulosus* from Thailand did not [39]. The ND5 protein structure of the two is also related to the aforementioned disparity. Therefore, the substantial decrease in transcript levels of *ND5* in *F. kawamurai* at all three temperatures observed in this study suggests that ND5 protein has a major role in regulating metabolism. Given the available data, the suppression of *ND5* transcript levels seems to be a sign that *F. kawamurai* is under thermal and cold stress. From the standpoint of protein function, Complex I is involved in the transmembrane transport of protons, which has to do with driving ATP generation [145]. Low- and high-temperature stress significantly lowered liver *ND5* gene transcript levels, which could lead to reduced oxidative phosphorylation coupling and proton leakage, lowering reactive ROS generation and regulating energy consumption [146,147,148]. Reducing proton leakage could also increase the efficiency of thermogenic nutrients into energy [37]. Consequently, taking into account the special features of *ND5* expression, it may be that ND5 is a key protein that regulates metabolism when mitochondria experience temperature change.

## 5. Conclusions

In this study, the mitochondrial genome of *F. kawamurai* from Guangzhou, Guangdong, was sequenced and its phylogenetic relationship was determined. To some extent, this species can link the frogs living in tropical or subtropical regions to mitochondrial gene expression levels at extreme temperatures. Under cold stress at 2 °C, *ATP8*, *ND1*, *ND2*, *ND3*, *ND4*, and *Cytb* gene transcript levels all increased substantially, whereas *ND5* significantly decreased. Under 4 °C stress, *COI*, *COII*, *COIII*, *ATP8*, *ATP6*, *ND2*, *ND4*, *ND4L*, *ND5*, and *ND6* gene transcript levels all decreased dramatically and significantly. In addition, under 40 °C stress, the transcript levels of *COIII*, *ND1*, *ND2*, *ND3*, *ND4*, *ND4L*, and *Cytb* genes increased considerably, whereas *COI* and *ND5* decreased significantly. The distinct metabolic states of organisms at different temperatures are predicted by the different levels of mitochondrial genome expression at different temperatures. In conclusion, significant differences in the expression levels of most mitochondrial PCGs in *F. kawamurai* exposed to low-temperature or high-temperature stress may mean that the frogs living in tropical or subtropical regions are highly susceptible to ambient temperature change. Further research into the impact of low- or high-temperature stress on mRNA transcript and protein levels in *F. kawamurai* will help to clarify the findings of this study.

## Figures and Tables

**Table 1:** Information about the samples used in this study and the NCBI GenBank accession numbers.

Family	Subfamily	Genus	Species	Genome Length	GenBank No.	References
Dicroglossidae	Dicroglossinae	*Limnonectes*	*Limnonectes bannaensis*	16,867 bp	AY899242	[38]
			*Limnonectes fujianensis*	18,154 bp	MF678821	[90]
			*Limnonectes fragilis*	16,640 bp	AY899241	Unpublished
		*Fejervarya*	*Fejervarya cancrivora*	17,843 bp	EU652694	[91]
			*Fejervarya kawamurai_GDGZ*	17,866 bp	OQ633008	This study
			*Fejervarya kawamurai*	17,650 bp	MH087466	[75]
			*Fejervarya limnocharis*	17,717 bp	AY158705	[79]
			*Fejervarya multistriata*	17,759 bp	MN987553	[92]
		*Hoplobatrachus*	*Hoplobatrachus rugulosus*	20,926 bp	JX181763	[93]
			*Hoplobatrachus tigerinus*	20,462 bp	AP011543	[94]
		*Phrynoderma*	*Phrynoderma hexadactylum*	20,280 bp	AP011544	[94]
		*Nanorana*	*Nanorana parkeri*	17,837 bp	KP317482	[95]
			*Nanorana pleskei*	17,660 bp	HQ324232	[96]
			*Nanorana ventripunctata*	18,373 bp	KY594708	[97]
			*Nanorana taihangnica*	17,412 bp	KJ569109	[98]
			*Nanorana yunnanensis*	23,685 bp	KF199150	[99]
		*Quasipaa*	*Quasipaa boulengeri*	17,741 bp	KC686711	[100]
			*Quasipaa verrucospinosa*	15,063 bp	KF199147	[99]
			*Quasipaa exilispinosa*	17,046 bp	MT561179	[101]
			*Quasipaa spinosa*	18,012 bp	FJ432700	[102]
			*Quasipaa jiulongensis*	15,072 bp	KF199149	[99]
			*Quasipaa shini*	14,943 bp	KF199148	[99]
			*Quasipaa yei*	17,072 bp	KJ842105	[103]
	Occidozyginae	*Occidozyga*	*Occidozyga martensii*	18,321 bp	GU177877	[104]

**Table 2:** *RT*-qPCR Primer of the 13 mitochondrial protein-coding genes in this study.

Gene Name	Forward Primers (5′-3′)	Reverse Primers (5′-3′)
*COI*	GDCC-COI-J TTGTTCACTGATTCCCACTTT	GDCC-COI-N GAGGTATCCCCGCTAAACCA
*COII*	GDCC-COII-J ATGGACGAGTTAGGTGCC	GDCC-COII-N AAGGTCATTTGTGGGGAT
*COIII*	GDCC-COIII-J GGCATCTACGGAACCACA	GDCC-COIII-N AAGCCGAAGTGGTGTTGA
*ATP8*	GDCC-ATP8-J ATGCCTCAATTACTACCT	GDCC-ATP8-N GCTTCAGGTTACAGAGTT
*ATP6*	GDCC-ATP6-J AATAAGTATTAACCTTCTCGG	GDCC-ATP6-N TACGGAGGCCGATAAGGACTG
*ND1*	GDCC-ND1-J CTTGCGGTAGCATTCCTCA	GDCC-ND1-N AGGATTTGCGAGGAGGTTG
*ND2*	GDCC-ND2-J TCAGGAGAATGGTCCATCG	GDCC-ND2-N ATGTTGAGAGGATTAGTCCA
*ND3*	GDCC-ND3-J CTCATTGCCTCTGCCCTA	GDCC-ND3-N GGAAGAAGCGTATGGAAT
*ND4*	GDCC-ND4-J GGCACTATTTTCCAACCC	GDCC-ND4-N AAGCAAGTAAAGAGGGAGTT
*ND4L*	GDCC-ND4L-J GGCCTATCTTTCCACCGTAT	GDCC-ND4L-N AAGGGGGATAGGACAAAAGA
*ND5*	GDCC-ND5-J TGCTGTGAAACACAACGACA	GDCC-ND5-N TGATTATTCCCGAGATTATGA
*ND6*	GDCC-ND6-J TTCTAATCCGTCACCATACT	GDCC-ND6-N TCCCACCTAAATACACTAGC
*Cytb*	GDCC-CYTB-J TCATCTAATCCAACAGGGCT	GDCC-CYTB-N GTGAAGTTATCTGGGTCTCC
*β-actin*	GDCC-Actin-J GTGCGTGACATCAAGGAG	GDCC-Actin-N GGCTTCTGGACATCTGAAC

**Table 3:** Location of features in the mtDNA of *F. kawamurai*.

Feature	Start Position	Stop Position	Intergenic Nucleotide	Length (bp)	Start Codon	Stop Codon	Anticodon	Strand
tRNA^Thr^	1	72	−1	72			TGT	H
tRNA^Pro^	72	140	17	69			TAG	H
tRNA^Leu(CUN)^	158	229	33	72			TGG	L
tRNA^Phe^	263	330		68			GAA	H
12S rRNA	331	1264	−1	934				H
tRNA^Val^	1264	1335		72			TAC	H
16S rRNA	1336	2927	−1	1592				H
tRNA^Leu(UUR)^	2927	2999		73			TAA	H
ND1	3000	3957		958	ATG	T		H
tRNA^Ile^	3958	4028		71			GAT	H
tRNA^Gln^	4029	4099	−1	71			TTG	L
tRNA^Met^	4099	4169	3	71			CAT	H
tRNA^Met^	4173	4241		69			CAT	H
ND2	4242	5276	−2	1035	ATT	TAG		H
tRNA^Trp^	5275	5343		69			TCA	H
tRNA^Ala^	5344	5412	2	69			TGC	L
tRNA^Asn^	5415	5487	2	73			GTT	L
tRNA^Cys^	5525	5590		66			GCA	L
tRNA^Tyr^	5591	5657	4	67			GTA	L
COI	5662	7192	11	1531	ATA	T		H
tRNA^Ser(UCN)^	7204	7274		71			TGA	L
tRNA^Asp^	7275	7342	2	68			GTC	H
COII	7345	8026		682	ATG	T		H
tRNA^Lys^	8027	8094	1	70			TTT	H
ATPase8	8096	8257	−7	162	ATG	TAA		H
ATPase6	8251	8932		682	ATG	T		H
COIII	8933	9716		784	ATG	T		H
tRNA^Gly^	9717	9785		69			TCC	H
ND3	9786	10,130	4	345	GTG	TAA		H
tRNA^Arg^	10,135	10,203	1	69			TCG	H
ND4L	10,205	10,483	−7	279	ATG	TAA		H
ND4	10,477	11,829	3	1353	ATG	TAA		H
tRNA^His^	11,833	11,901		69			GTG	H
tRNA^Ser(AGY)^	11,902	11,969	259	68			GCT	H
ND6	12,229	12,717	5	489	ATG	AGG		L
tRNA^Glu^	12,723	12,790	5	68			TTC	L
Cytb	12,796	13,932		1137	ATG	TAA		H
D-loop	13,933	16,039		2107				H
ND5	16,040	17,857		1818	GTA	TAA		H

Notes: “H” means gene encoded by the H-strand; “L” means gene encoded by the L-strand. Intergenic nucleotide represents a noncoding base between genes; a negative number (−) denotes a gene overlapping.

**Table 4:** The base composition of the mitochondrial genomes of *F. kawamurai*.

Region		A (%)	T (%)	C (%)	G (%)	A + T (%)	C + G (%)	AT Skew	GC Skew
Mito (H strand)		27.8	29.4	27.5	15.4	57.2	42.9	−0.028	−0.282
PCGs	J	25.3	31.2	28.7	14.7	56.5	43.4	−0.104	−0.322
	N	17.2	34.8	12.1	36.0	52.0	48.1	−0.339	0.498
tRNAs (H strand)		29.3	27.1	23.4	20.2	56.4	43.6	0.039	−0.072
rRNAs (H strand)		33.3	24.4	23.7	18.6	57.7	42.3	0.154	−0.119

## Data Availability

The data supporting the findings of this study are openly available from the National Center for Biotechnology Information at https://www.ncbi.nlm.nih.gov (accessed on 17 May 2023). The accession number is OQ633008.

## Supplementary Materials

The following supporting information can be downloaded at: https://www.mdpi.com/article/10.3390/ani13193015/s1, Figure S1. The secondary structure of 23 tRNAs in *F. kawamurai* mitochondrial genome; Table S1. The partition schemes and best-fitting models selected.

## Abbreviations

Mitogenome	mitochondrial genome
PCR	polymerase chain reaction
NCBI	National Center for Biotechnology Information;
bp	base pair
PCGs	protein-coding genes
CRs	control regions
BI	Bayesian inference
ML	maximum likelihood.
