# Supplementary material for: How Does Mitochondrial Protein-Coding Gene Expression in Fejervarya kawamurai (Anura: Dicroglossidae) Respond to Extreme Temperatures?
Source: Animals (Basel). 2023 Sep 25;13(19):3015. doi: 10.3390/ani13193015 (PMC10571990; doi:10.3390/ani13193015)
Supplement: Supplementary file 1 [file animals-13-03015-s001.zip › Table S1. The partition schemes and best-fitting models selected.pdf]

Table S1. The partition schemes and best-fitting models selected.

Nucleotide Sequence Alignments		
Subset	Subset Partitions	Best Model
Partition 1	ND3_pos1, ND1_pos1, ND4L_pos1, ATP6_pos1, ATP8_pos1, ND2_pos1, ND4_pos1	GTR+I+G
Partition 2	ND3_pos2, ND1_pos2, ATP6_pos2, ATP8_pos2, ND2_pos2, ND4L_pos2, ND4_pos2	TVM+I+G
Partition 3	ND2_pos3, ND4_pos3, ND4L_pos3, ATP6_pos3, ATP8_pos3	TRN+I+G
Partition 4	COII_pos1, Cytb_pos1, COIII_pos1, COI_pos1	SYM+I+G
Partition 5	COI_pos2, Cytb_codon2, COIII_codon2, COII_pos2	HKY+I+G
Partition 6	COI_pos3, ND3_pos3, ND1_pos3, COIII_pos3, COII_pos3	TIM+I+G
Partition 7	Cytb_pos3	TRN+I+G
Partition 8	ND6_pos1, ND6_pos2	HKY+G
Partition 9	ND6_pos3	TRN+G

Note: The full names of all abbreviations are as follows: pos1: first codon; pos2: second codon; GTR: general time reversible; I: unchanged site proportion; G: Gamma distribution.
