# Supplementary figures and images for: How Does Mitochondrial Protein-Coding Gene Expression in Fejervarya kawamurai (Anura: Dicroglossidae) Respond to Extreme Temperatures?
Source: Animals (Basel). 2023 Sep 25;13(19):3015. doi: 10.3390/ani13193015 (PMC10571990; doi:10.3390/ani13193015)

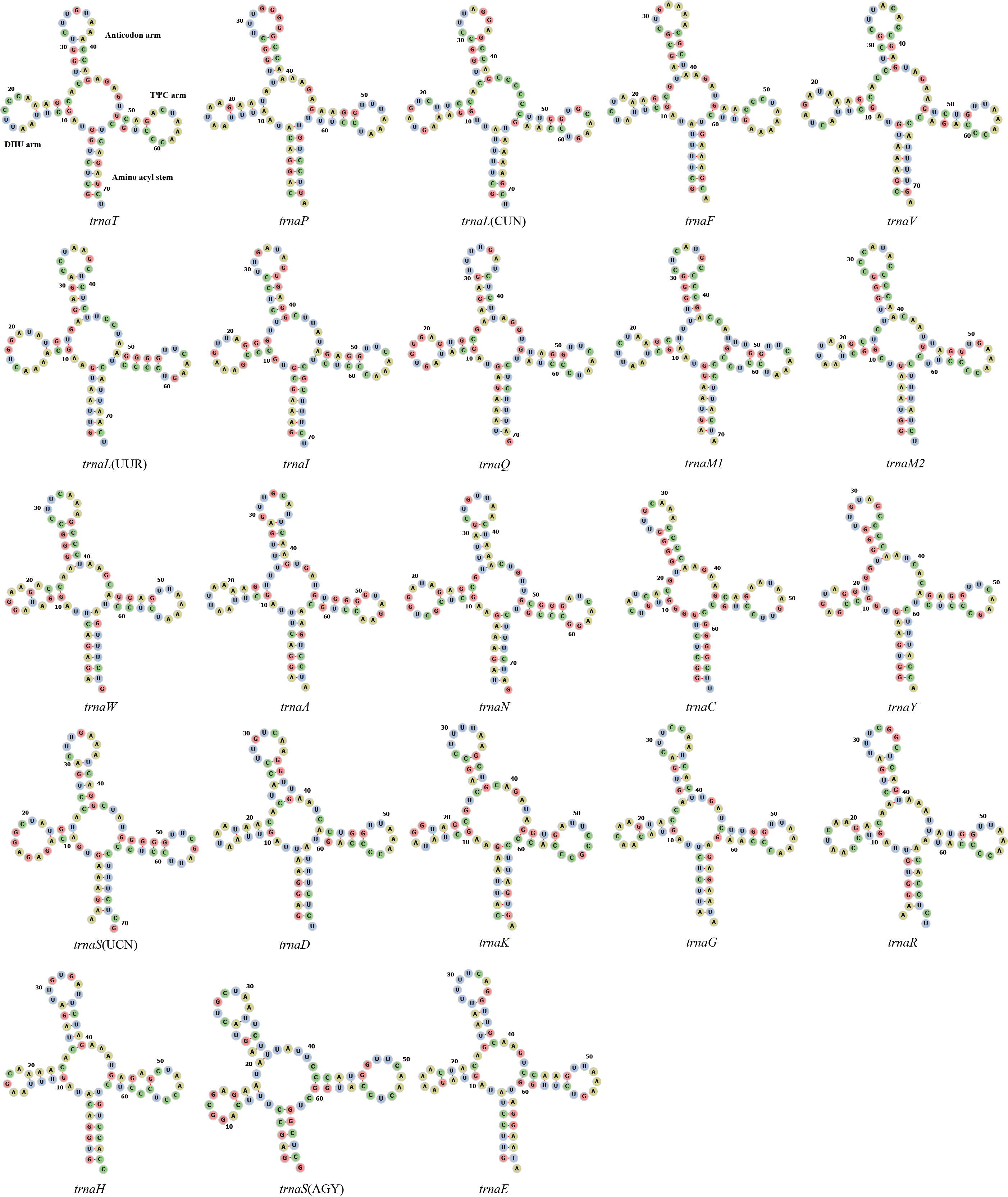

Supplement: Supplementary file 1 [file animals-13-03015-s001.zip › Figure S1. The secondary structure of 23 tRNAs in F. kawamurai mitochondrial genome.png]
